# An Unusual Cause of Altered Mental Status in Elderly—Acute Cerebellitis: A Case Report and Review

**DOI:** 10.1155/2013/653925

**Published:** 2013-12-09

**Authors:** Priyank Patel, Supratik Rayamajhi, Hemasri Tokala, Heather Laird-Fick

**Affiliations:** Department of Internal Medicine, B-301 Clinical Center, Michigan State University, East Lansing, MI 48824, USA

## Abstract

Acute cerebellitis is a rare diagnosis found mostly in the pediatric population. The etiology, in most instances, is unknown. We describe the case of a 61-year-old woman who presented with acute mental status changes, signs of cerebellar dysfunction, and MRI findings of acute cerebellitis. A brief review of the existing literature and comparison of our case with previous reports are also presented.

## 1. Introduction

Acute cerebellitis (AC) is a rare inflammatory syndrome characterized by rapid onset of cerebellar dysfunction, usually without an obvious etiology. It is often used with the term “acute cerebellar ataxia” which is appropriately described as syndrome of cerebellar dysfunction; however there exists some overlap due to unclear mechanisms of underlying pathophysiology [1]. There has been a debate about its progression, since both a benign and a fulminant course have been described in previously reported cases. Complications such as tonsillar herniation, hydrocephalus, and severe cerebellar atrophy can occur. The majority of the cases have been reported in the pediatric population, with extremely few adult cases. We describe the case of a 61-year-old woman who presented with acute mental status changes, signs of cerebellar dysfunction, and MRI findings of AC.

## 2. Case Report

A 61-year-old female was brought to the emergency department with an altered mental status. The neighbors notified emergency services (EMS) when they did not see her for 2 days. She was found in a confused state, lying on the kitchen floor. She was hemodynamically stable at the time of initial evaluation. Past medical history included osteoarthritis of the knees and alcohol dependence for the last 2 years. Her family history was noncontributory. She was admitted for alcohol detoxification 10 months prior to this event. On exam, her temperature was 98.3 F, blood pressure was 108/58 mm Hg, heart rate was 98 beats per minute, and respiratory rate was 18 per minute. She was awake but lethargic and disoriented to time, place, and person. Her gait was unsteady and she was not able to walk without assistance. Detailed neurological exam was notable only for bilaterally positive finger-to-nose test and intentional tremor of the upper extremities. Romberg test was not assessed due to her unsteadiness.

Her initial blood work revealed a serum sodium of 115 mEq/L, potassium of 3 mEq/L, serum, serum magnesium magnesium of 1 mEq/L, and an anion gap of 20 other lab parameters, including complete blood count, urine drug screen, blood ethanol level and liver function testes were essentially normal. A brain CT showed age-appropriate atrophic changes. A lumbar puncture was not performed due to unclear reasons, most likely being code status precluding invasive procedures.

**Figure 1:**
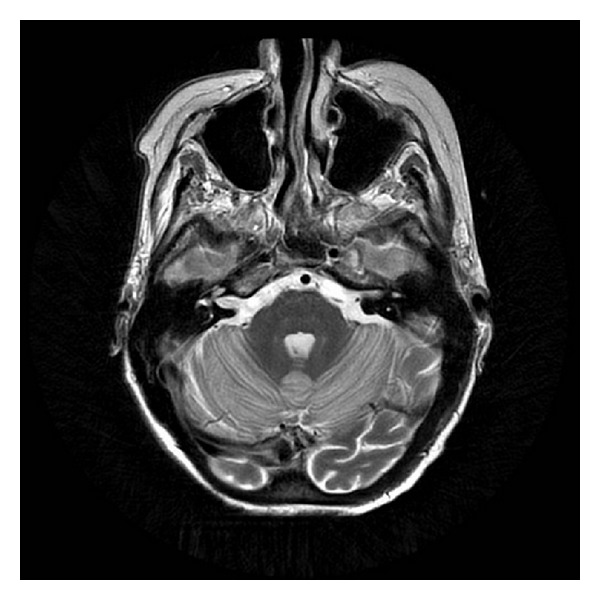
Magnet resonance imaging: axial T2-weighted images showing increased signal intensity in cerebellar hemispheres.

**Figure 2:**
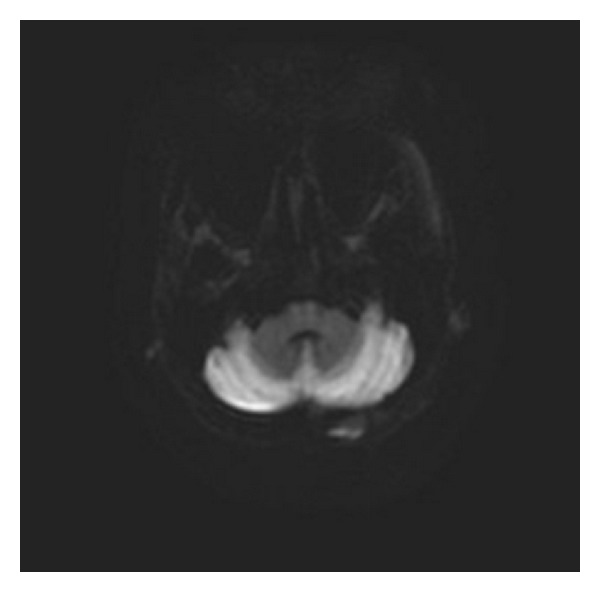
Magnet resonance imaging: diffusion image showing abnormal signal intensity in the cerebellum.

**Figure 3:**
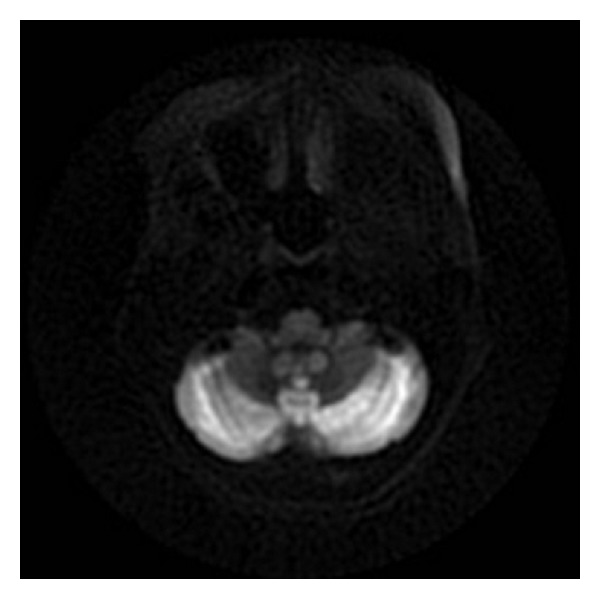
Magnet resonance imaging: diffusion propeller image with abnormal signal intensity in the cerebellum.

She was admitted to the hospital and adequately hydrated. On the second day of admission, her speech became garbled and she became more somnolent. Her temperature dropped to 94.8 F and was immediately transferred to the intensive care unit. The hypothermia was gradually reversed by forced warm air therapy using a Bair Hugger blanket. Over time, the patient became more somnolent and lethargic. She remained disoriented and became completely aphasic. She was also noted to have developed difficulty in swallowing her food. MR images of the brain showed diffusely abnormal signal within the cerebellum. The cerebellar cortex showed low signal on T1 and symmetrically increased signal intensity on T2-weighted images (Figure 1). There was increased signal on diffusion sequence throughout the cerebellar cortex (Figures 2 and 3). The radiologist interpreted this as “acute cerebellitis.” Further investigations to exclude alcohol related vitamin deficiencies were normal. Vitamin B12 levels of 749 pg/ml (211–911 pg/ml), folate of 4.76 ng/ml (<3.38 ng/ml considered deficient), thiamine of 129 nmol/L (80–150 nmol/L), copper of 1.03 mcg/mL (0.75–1.45 mcg/ml), and zinc 0.54 mcg/ml (0.66–1.10 mcg/ml). Peripheral blood herpes simplex virus (HSV) PCR was negative. Other viral panels were not obtained. Blood cultures during the admission were negative for any growth.: Although her hypothermia was successfully reversed, her altered mental status remained unchanged. She continued to be aphasic and dysphagic. After about a week of little or no improvement in her mental status, she finally began to verbalize—though confabulatory—while remaining oriented only to self.

She had a protracted hospital stay, spanning 12 weeks, albeit mostly over placement concerns. She gradually became more alert and coherent and was able to recognize the staff members who visited her frequently. She continued to require support during ambulation due to persistent gait instability. She failed multiple swallow evaluations despite aggressive speech therapy and ultimately a percutaneous gastrostomy tube was placed to facilitate enteral nutrition. She was eventually transferred to an extended care nursing home facility.

## 3. Discussion

Acute cerebellitis (AC) remains an unclear clinical entity that has been associated with multiple etiologies including viruses (e.g., varicella zoster virus (VZV), measles, mumps, rubella, Epstein-Barr virus (EBV), cytomegalovirus, herpes simplex virus, parainfluenza virus, poliovirus, and coxsackie virus) and bacteria (e.g., *Salmonella typhi*, *Borrelia burgdorferi*, *Coxiella burnetii*, *Bordetella pertussis*, and *Mycoplasma pneumoniae*), occurring either as a primary infectious or postinfectious process or after vaccination against some of these pathogens [2–4]. EBV and VZV appear to be the most frequent pathogens associated with AC [1]. Most of the published cases occurred in the pediatric population. Very few cases have been reported in adults and in most of these instances the etiology remained unclear. Few reported cases of adult cerebellitis are summarized in Table 1.

The most frequent clinical features of AC are headache, vomiting, lethargy, altered mental status, coma, ataxia, and fever. Our patient presented with predominant symptoms of mental status changes and gait ataxia and developed dysphagia much later in her course.

There are varying descriptions of the course of AC in the reported literature, ranging from benign and self-resolving to a complicated course with hydrocephalus, tonsillar herniation [4], and cerebellar atrophy late in the course of disease [11]. Hydrocephalus and tonsillar herniation are most likely to be complications of the acute phase and may require neurosurgical intervention to prevent death [12]. Our patient had experienced hypothermia, altered mental status, aphasia- and dysphagia but did not have any life-threatening complications. There have been reported cases of aphasia following cerebellar damage [12]. In this case, acute cerebellitis caused cerebellar damage causing aphasia followed by residual dysarthria that did not resolve during the 12-week hospitalization. The cerebellar inflammation also caused gait instability and dysphagia in this patient.

## 4. Conclusion

AC is a poorly understood clinical entity with uncertain etiology and heterogeneous pathogenesis. The presentation of the disease, however, can be somewhat similar in many cases, with altered mental status, headache, vomiting, and gait ataxia in both adults and children. MRI is the diagnostic modality of choice as CT may not detect the posterior fossa inflammatory process. Worsening clinical symptoms should warrant repeated imaging to promptly identify life-threatening complications that may require neurosurgical intervention. Anecdotal evidence suggests some role of steroids and antimicrobials, especially when etiology is identifiable. AC is rare in adults and the standards of management for this condition are not currently established—largely due to an inability to identify a clear etiological factor.

## Figures and Tables

**Table 1:** Reported cases of cerebellitis in adults.

Author	Patient	Etiology	Principal symptoms	MRI findings	Outcome
Flanagan et al. [5] (2013)	41 M	Unknown, patient with Crohn's disease	Headache and ataxia and cerebellar signs	T2 signal abnormalities, pial enhancement and cerebellar enlargement	Alive
Rizek et al. [6] (2013)	63 F	*Salmonella typhimurium* (blood culture)	Meningeal signs, appendicular ataxia	Diffuse cerebellar hyperintense signal on T2 sequence	Alive with residual dysarthria and dysmetria
Ishikawa et al. [7]	25 F	*Influenza A *(H3N2)	Dysarthria, slurred speech, limb and truncal ataxia	High signal intensity in the cerebellar cortex on T2-weighted MRI	Alive with partial neurological recovery
Sugiyama et al. [8] (2000)	35 F	Unknown	Fever, headache, stiff neck, coma	Diffuse cerebellar cortical, T2 high signal, pontine lesion	Alive, complete recovery
Ravi and Rozen [9] (2000)	22 F	Unknown	Ataxia, fever, headache, stiff neck	Diffuse cerebellar swelling, hydrocephalus, leptomeningeal enhancements	Alive with improvement in symptoms
Bakshi et al. [10] (1998)	21 M	Unknown	Vomiting, headache	Diffuse cerebellar swelling, herniation of the tonsil, leptomeningeal enhancement	Alive with minimal residual neurological deficits at 1 year
